# Human XIRP1 is a new podosome protein targeting cytosolic bacteria as part of the IFN-γ defense program

**DOI:** 10.1093/jimmun/vkag116

**Published:** 2026-06-15

**Authors:** Rodolfo Urbano, Alexander S Low, Aglaia Ntokou, Eui-Soon Park, Kyle Tretina, Alexandru Tunaru, Ryan G Gaudet, John D MacMicking

**Affiliations:** Howard Hughes Medical Institute, Chevy Chase, MD, United States; Yale Systems Biology Institute, West Haven, CT, United States; Department of Microbial Pathogenesis, Yale University School of Medicine, New Haven, CT, United States; Department of Pathology, Microbiology and Immunology, University of California Davis School of Veterinary Medicine, Davis, CA, United States; Department of Pathology, Microbiology and Immunology, University of California Davis School of Veterinary Medicine, Davis, CA, United States; Department of Pathology, Microbiology and Immunology, University of California Davis School of Veterinary Medicine, Davis, CA, United States; Howard Hughes Medical Institute, Chevy Chase, MD, United States; Yale Systems Biology Institute, West Haven, CT, United States; Department of Microbial Pathogenesis, Yale University School of Medicine, New Haven, CT, United States; Department of Immunobiology, Yale University School of Medicine, New Haven, CT, United States; Howard Hughes Medical Institute, Chevy Chase, MD, United States; Yale Systems Biology Institute, West Haven, CT, United States; Department of Microbial Pathogenesis, Yale University School of Medicine, New Haven, CT, United States; Department of Immunobiology, Yale University School of Medicine, New Haven, CT, United States; Howard Hughes Medical Institute, Chevy Chase, MD, United States; Yale Systems Biology Institute, West Haven, CT, United States; Department of Microbial Pathogenesis, Yale University School of Medicine, New Haven, CT, United States; Department of Immunobiology, Yale University School of Medicine, New Haven, CT, United States; Howard Hughes Medical Institute, Chevy Chase, MD, United States; Yale Systems Biology Institute, West Haven, CT, United States; Department of Microbial Pathogenesis, Yale University School of Medicine, New Haven, CT, United States; Department of Immunobiology, Yale University School of Medicine, New Haven, CT, United States; Department of Microbiology and Immunology, Columbia University, New York, NY, United States; Howard Hughes Medical Institute, Chevy Chase, MD, United States; Yale Systems Biology Institute, West Haven, CT, United States; Department of Microbial Pathogenesis, Yale University School of Medicine, New Haven, CT, United States; Department of Immunobiology, Yale University School of Medicine, New Haven, CT, United States

**Keywords:** actin, cell-autonomous immunity, interferon, podosomes

## Abstract

Interferon-gamma (IFN-γ) is a powerful transactivating signal eliciting hundreds of IFN-stimulated genes (ISGs) in humans to help combat infection. Most ISGs remain uncharacterized, and here we searched for actin-binding candidates that could potentially target intracellular pathogens to block their spread or promote immune cell migration into infected tissues. Dual RNA-Seq and in silico mining across 1,933 data sets discovered >225 actin-related genes; the most highly expressed was XIRP1 (xin actin binding repeat containing 1 protein), a new ISG with no reported immune function. We found XIRP1 induction required IFN-γ plus IL-1β or exposure to pathogenic *Listeria, Shigella,* or *Salmonella* in immune and non-immune cells. Within IFN-γ-activated human macrophages, the XIRP1 protein localized to actin-rich podosomes where it formed a dome-shaped cap facing the cytosol; genetic XIRP1 ablation led to significant actin loss from these structures. Within infected cells, XIRP1 was recruited onto cytosolic *Listeria monocytogenes* in an ActA-dependent manner. Live imaging found many listeriae were fully encapsulated by XIRP1 whereas incomplete XIRP1 coating allowed pathogen escape from the initial coat structure. Together, our results identify XIRP1 as a new podosome-associated ISG that targets cytosolic bacteria as part of the IFN-γ-induced defense program in humans.

## Graphical abstract

**Graphical Abstract:**
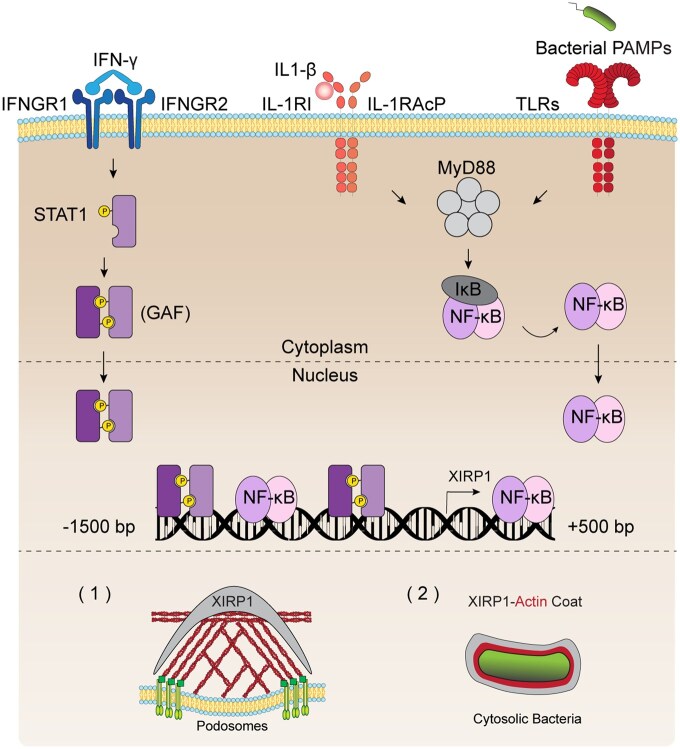

## Introduction

Cell-autonomous immunity is an ancient defense strategy deployed by all living organisms to combat infection.^1–3^ In vertebrates, this defensive strategy is often mobilized by signals from the IFN family of cytokines that elicit hundreds of ISGs—sometimes termed the “interferome”—in both immune and non-immune cells.^1^^,^^4^^,^^5^ Given the breadth of the human IFN response, it is not surprising that many ISGs remain poorly characterized or lack any ascribed function.^1^^,^^6^ Recent large-scale efforts using CRISPR-Cas9 or over-expression screens have met with some success, helping unearth new ISGs that restrict pathogenic bacteria, parasites, or viruses.^7–10^ Other approaches including AI-driven computational screens may offer an additional pipeline for discovery.

Here, we used in silico data mining and genome-wide transcriptomic approaches to probe for actin-associated ISGs involved in the immune response to bacterial infections.^11^ Recent evidence implicates human ISGs in actin-related host defense. IFN-γ-inducible guanylate binding proteins (GBPs), for example, target actin-utilizing pathogens to immobilize them or build signaling platforms that trigger downstream inflammasome and bactericidal activities.^8^^,^^12–16^ Furthermore, IFN-γ is known to affect actin polymerization and macrophage motility, the latter of which depends on an actin-rich podosome network.^17–19^ However, the factors regulating actin dynamics following IFN-γ stimulation remain unclear. In the current manuscript, we identify XIRP1 (xin repeat actin-binding protein 1) as a new ISG that encapsulates actin-rich podosomes and actin-utilizing bacteria as part of the IFN-γ defense program inside human cells.

## Materials and methods

### Cell lines and growth conditions

Cell cultures were maintained at 37 °C in a 5% CO_2_ incubator. Normal human fibroblast Hs27 cells (ATCC, CRL-1634) were cultured in DMEM (Thermo Fisher Scientific) supplemented with 10% heat-inactivated fetal bovine serum (FBS) (Thermo Fisher Scientific). Primary human intestinal myofibroblast (Lonza Group AG, CC-2902) were maintained in SmGM Medium with growth factors (Lonza Group AG). Human THP-1 monocytes (ATCC, TIB-202) were grown in RPMI 1640 Medium with ATCC modification (Thermo Fisher Scientific) supplemented with 10% FBS and 50 µM 2-mercaptoethanol (Millipore Sigma). Macrophages were generated by differentiating THP-1 monocytes with 100 ng/ml phorbol 12-myristate 13-acetate (PMA) (Millipore Sigma, P8139). Macrophages in podosome experiments were differentiated for 24 h, PMA was removed, and then cytokines added for an additional 24 h. For bacterial infections, macrophages were differentiated with PMA for 48 h, PMA was removed, and cytokines added for an additional 24 h. Unless otherwise indicated, cells were treated with either 10 ng/mL IFN-β (Thermo Fisher Scientific), 50 ng/ml IFN-γ (R & D Systems), 10 ng/ml IL-1β (R & D Systems),100 ng/ml IL-6 (R & D Systems), 100 ng/ml MCP-1 (R & D Systems), or a combination of cytokines.

### RNA-Seq analysis and large-scale data mining

Hs27 cells were cultured in DMEM (10% FBS, 2 mM glutamine) to ∼70 − 80% confluency in T-175 flasks. Duplicate samples were either left untreated or treated with 50 ng/ml IFN-γ for 24 h. Cells were harvested using 0.25% trypsin/EDTA (Thermo Fisher Scientific, 25200056), and RNA was isolated using the Direct-zol RNA miniprep kit (Zymo Research, R2051). Total RNA was sent to GENEWIZ for standard library preparation, PolyA selection, and RNA sequencing (Illumina HiSeq, 2 × 150 bp).

To mine macrophages RNA-seq data sets, an R-script downloaded from the ARCHS^4^ website was used to generate a matrix containing gene-level counts from 1,933 macrophage datasets available from the Sequence Read Archive (SRA).^20^ Quantile normalization was performed using Bioconductor (biocLite) and the preprocessCore package. Normalized XIRP1 expression was plotted with GraphPad Prism and M1-polarized THP-1 macrophage data sets (GEO accession: GSM3729256-8) were identified based on high XIRP1 expression (top 1.5% of all data sets). Raw fastq files from the SRA were downloaded along with files from control THP-1 monocyte samples (GEO accession: GSM3729250-2).

The Farnam high performance computer cluster from the Yale Center for Research Computing was used for RNA-seq data analysis following a pipeline from rnabio.org.^21^ Briefly, sequencing adapters were trimmed with Flexbar and quality of reads evaluated with FASTQC; reads were aligned using HISAT2 and read counts generated with HTSEQ; differential expression analysis and statistical significance were performed with edgeR using the exact-test and adjusting *P-values* based on a 5% false discovery rate. Volcano plots of DEGs were generated with GraphPad Prism. Plots exclusively containing actin-associated genes were filtered with the BioVenn web application based on Gene Ontology Terms (GO: 0015629, GO: 0003779, GO: 0005884, GO: 0031941, GO: 0045010, GO: 0007014).

### Phylogenetics and other bioinformatic tools

An alignment containing amino acid sequences from 221 XIRP1 homologues was generated using the online *MAFFT* FFT-NS-i algorithm (mafft.cbrc.jp/alignment/server). A maximum likelihood tree with a JTT matrix-based model was constructed using *MEGA* version 10.1.8.^22^^,^^23^ For clarity, tree branches were collapsed among closely related species. Protein domains were analyzed with *NCBI Orthologs*, the *Conserved Domain Database*, and the *Conserved Domains-Search* tool (NCBI). Gene synteny analysis within the ± 1,000,000 base pair region flanking XIRP1 was performed with the NCBI Genomic Context browser. Immunity-related genes (eg MYD88, CX3CR1, CCR8) were identified within the human XIRP1 region and presence or absence of genes was determined in representative organisms from mammals (*P. troglodytes, M. musculus, O. anatinus*), birds (*D. novaehollandiae*), reptiles (*A. mississippiensis*), amphibians (*M. unicolor, X. laevis*), fish (*D. rerio, L. oculatus, C. milii*), and sea lamprey (*P. marinus*).

Predicted binding sites for transcription factors were identified through the *TF Analysis* online tool (interferome.org). Independent verification was done through the Swiss Institute of Bioinformatics *Eukaryotic Promoter Database* (epd.epfl.ch).

### Bacterial strains and growth conditions

Strains for cell culture experiments included GFP-expressing *Listeria monocytogenes* 10403S (DHL1039), *actA* and *hly plcAB* mutants (H. Agaisse). *Listeria* was grown overnight in brain heart infusion (BHI) broth (HiMedia Laboratories, LLC) at 28 °C with shaking (225 rpm). *Shigella flexneri* M90T (F. Randow) and *Salmonella* Typhimurium 1344 (J. Galan) expressing GFP from pFPV25.1 (Addgene) were grown in Luria-Bertani (LB) broth (Thermo Fisher Scientific) containing carbenicillin (100 µg/ml) at 37 °C overnight with shaking (250 rpm). For fibroblast infections, *Shigella* was sub-cultured in fresh LB (1:100) and grown to OD_600_ = 0.6 at 37 °C with shaking (250 rpm). *Salmonella* was sub-cultured to OD_600_ ∼1.0.

### Construction of plasmids

For CRISPR-knockout plasmids, 2 oligonucleotide pairs carrying sgRNA sequences were designed to target XIRP1 and ordered (Integrated DNA Technologies) for cloning into pSpCas9(BB)-2A-Puro (pX459) V2.0 (Addgene, 62988). A pair was designed targeting a region upstream of the translational start site and consisted of 5′-CACCGGG AGTGACCATGGTACCAC-3′ and 5′- AAACGTGGTACCATGGTCACTCCC-3′. The second pair was designed to target downstream of the translational stop site and consisted of 5′- CACCGATTCACACATGTCGATGCGT-3′ and 5′-AAACACGCATCGACATGTGTGAATC-3′. Oligonucleotide pairs (10 µM) were hybridized in T4 Ligase buffer using a thermocycler where samples were incubated at 95 °C for 5 min, 95 °C for 15 s, and 92.5 °C for 15 s. The last two incubations were repeated for 28 cycles, lowering the temperature by 2.5 °C every cycle. Plasmid pX459 was digested with *Bbs*I (New England Biolabs) and purified with the Wizard SV Gel and PCR Clean-Up System (Promega) after DNA electrophoresis. Ligation of cut pX459 (100 ng) and diluted hybridized oligonucleotides (1:200) was performed with T4 Ligase in 20 µl reactions by incubating for 30 min at room temperature. Plasmid constructs were electroporated into *E. coli* DH10B ElectroMAX (Thermo Fisher Scientific) bacteria and verified by Sanger sequencing (Genewiz, Inc).

Retroviral vectors introducing individual XIRP1 isoforms were generated with pMSCVpuro (Clontech Laboratories, 631461). Since the XIRP1 coding region is entirely within exon 2, PCR amplification was directly done from gDNA. Isoform A was amplified with primer 5′-gaattagatctctcgaggttCCACCATGGCCGACACCCAGACACAGGTG-3′ and 5′-cctacccggtaga attcgttTCACTGGGCAGCTGGCTGGGAGTAGCTGCA-3′; isoform B with 5′-gaattagatctctcgag gttCCACCATGGCCGACACCCAGACACAGGTG-3′ and 5′-ctacccggtagaattcgttTCACAGCA GCTTTCTGGGGGCTGCTGGGATCCGGGGATCAC-3′; isoform C with 5′-gaattagatctctcgagg ttCCACCATGCCCCCAAAGAAGAAGCCGCAGCTG-3′ and 5′-cctacccggtagaattcgttTCACTG GGCAGCTGGCTGGGAGTAGCTGCA-3′. Fragments were cloned into *Hpa*I-digested pMSCVpuro using the Gibson Assembly Cloning Kit (New England Biolabs, E5510S). Plasmids were transformed into *E. coli* NEB 5-alpha and the purified plasmids verified by Sanger sequencing (Genewiz, Inc).

To construct the XIRP1-mCherry translational reporter, the retroviral vector pMSCV-IRES-mCherry FP (addgene, 52114) was modified by fusing the sequence from XIRP1 (isoform b) to the N-terminus of mCherry. The XIRP1 (isoform b) fragment was PCR amplified with a C-terminal linker (GlyGlyGlyGlySer) added with primers 5′-CACCATGGCCGACACCCAGACA CAG-3′ and 5′-GGAGCCCCCTCCGCCCTTTCTGGGGGCTGCTGGGATC-3′. To allow for Gibson cloning, a second PCR was performed adding complementary regions to the vector with primers 5′-tttgaaaaacacgataatacCACCATGGCCGACACCCAG-3′ and 5′-tcctcctcgcccttgctcacG GAGCCCCCTCCGCCCTT-3′. The resulting fragment was then incorporated to *Nco*I-digested pMSCV-IRES-mCherry using the Gibson Assembly Cloning Kit (New England Biolabs, E5510S). The construct was transformed into *E. coli* NEB 5-alpha and the purified plasmid verified by Sanger sequencing (Genewiz, Inc).

### CRISPR-Cas9 mutagenesis

Gene-knockout in Hs27 fibroblasts was performed by transfecting pX459 derived plasmids with WI-38 Cell Avalanche Transfection Reagent (EZ Biosystems, LLC). A day prior to transfections, cells were seeded in DMEM (10% FBS) at 2 × 10^5^ cells per well in 6-well plates. The two plasmids delivering sgRNAs targeting XIRP1 were mixed 1:1 and 4 mg of total endotoxin-free DNA was added to 200 µl OptiMEM containing 6.4 µl of transfection reagent. The mixture was briefly vortexed, incubated for 15 min at room temperature, and 150 µl was added per well. Plates were centrifuged at 300×*g* for 5 min, then incubated for 5 h at 37 °C in a 5% CO_2_ incubator. The media was replaced to remove transfection reagent, and puromyocin (1 − 2 µg/ml) selection was started 24 h later. Selection was held for 24 − 48 h and media was then replaced with DMEM (10% FBS) without puromycin to allow cells to recover for 24 − 48 h. Adherent cells were dislodged with StableCell Trypsin Solution (Millipore Sigma) and diluted (∼3 cells per 200 µl) in fresh DMEM (20% FBS) containing 33% spent media from Hs27 cultures (80%−95% confluent). Clone isolation was performed by limited dilution in 96-well plates using 200 µl of cell suspension per well. The media was replaced every fifth day for ∼25 d or until clones were clearly visible using 4x magnification. To screen clones, DNA extract was prepared from an aliquot of cells with QuickExtract DNA Extraction Solution (Lucigen Corporation). Only wells containing single clones were screened using PCR primers flanking the Cas9 cut sites. Cut site 1 was screened using PCR primer pair 5′- CTAGCTCAGACATTCTCAGTC-3′ and 5′-GGTAGTAAACAGGGCTCA G-3′; the second cut site was screened with 5′-CAGGACTGAAGTGGGTAC-3′ and 5′- GGAACACATACTGGAACAGATG-3′. The absence of PCR product with primers flanking both cut sites, and positive PCR signal with primer pairs within exon 2, suggests gene inactivation in the HS27 XIRP1-knockout clone resulted from a DNA inversion event.

As THP-1 monocytes are difficult to transfect, gene-knockout was performed by electroporation of TrueCut Cas9 Protein V2 (Thermo Fisher Scientific) complexed to TrueGuide Synthetic guide RNAs (Thermo Fisher Scientific). Gene-disruption was performed with two sgRNAs, one targeting upstream of the translational start site and another within Exon 2. Sequences of the 1-piece chemically modified synthetic gRNAs were 5′-GGGAGUGACCAUGGUAC-3′ and 5′-GAGACGUUCGUGCAGCCCGC-3′. A total of 20 µg Cas9 was mixed with 120 pmoles of each sgRNA in a 6 µl volume and incubated for 10 min at room temperature. Complexed Cas9-sgRNA was added 49 µl of BTXpress electroporation solution (BTX Molecular Delivery Systems) and incubated for 10 min at room temperature. Media was removed from THP-1 monocytes cultures (5 − 6 × 10^5^ cells/ml) and cells were washed once with DPBS (Thermo Fisher Scientifics). A total of 2 × 10^6^ cells were resuspended in 50 µl of BTXpress solution and the Cas9-sgRNA complex was added. A 100 µl aliquot of the mixture was transferred to a 0.2 cm gap cuvette and electroporation was performed at room temperature using a square wave protocol (140 V, 1 pulse of 10 msec; field strength 750 V/cm) with an ECM 830 Square Wave Electroporation System (BTX Molecular Delivery Systems). Mouse T cell Nucleofector media (100 µl) was added and cells were immediately transferred to a 24-well culture dish containing 1 ml of additional media. Cells were cultured for 72 h prior to plating for clone isolation. Limited dilution was performed as with Hs27 cells, but 96-well round bottom plates were used instead of flat bottom, and cells were diluted in RPMI media containing 33% spend media from THP-1 suspension cultures (density 6 − 8 × 10^5^ cells/ml). Clones were PCR-screened for deletions with primers 5′-CTAGCTCAGACATTCTCAGTC-3′ and 5′-GTCTCAAA GATCCAGCGAG-3′ and verified by Sanger sequencing (Genewiz, Inc) with 5′- CTTCACTGTC TTTTTCACATCAC-3′ and PCR primers. Sequence from the XIRP1-knockout clone showed an 862 bp deletion occurring between the two sgRNA targeting sites.

### Fibroblast and macrophage infections

Hs27 fibroblast cells were seeded in 4-well chambered cover glass (Cellvis, C4-1.5H-N) at 0.6 × 10^5^ cells per chamber in 0.5 ml DMEM. Media was replaced with DMEM containing 50 ng/ml IFN-γ or without cytokine and cells were incubated for 24 h. *Listeria* were grown as previously indicated, 1 ml of 18 − 24 h overnight culture was centrifuged at 10,000 × g for 3.5 min, washed once with phosphate buffer saline (PBS) (Thermo Fisher Scientific), then resuspended at ∼2.5 × 10^9^ CFU/ml in pre-warmed DMEM. Serial dilutions (1:1,600, 1:3,200, 1:6,400) were prepared and fibroblast monolayers were infected with 0.5 ml of diluted bacteria (MOI ∼ 13, 6.5, 3.3). Chambered cover glass was centrifuged at 1,000 × g for 5 min prior to incubating for 1 h at 37 °C in a 5% CO_2_ incubator. To remove extracellular bacteria, chambers were washed twice with DMEM and media containing 10 µg/ml gentamycin was added. Bacterial infection foci were allowed to develop for 24 h prior to fixation with 4% paraformaldehyde (PFA) solution (Santa Cruz Biotechnology, Inc) for 20 min. *Shigella* infections (MOI ∼ 5.7, 1.1, 0.2) were performed similarly with serial dilutions prepared from OD_600_ = 0.6 cultures. Primary intestinal myofibroblast treated with indicated IFN-γ concentrations were infected with *Salmonella* (MOI ∼20) prepared from OD_600_ ∼1.0 cultures.

### Immunoblots

Fibroblasts were seeded (∼2.5 × 10^5^ cells/well) in 6-well plates containing 2 ml DMEM and allowed to adhere for 24 h or until ∼80%−90% confluent. Media was replaced with DMEM containing indicated cytokines and cells incubated for 24 h. For THP-1 cells, monocytes (6 × 10^5^ cells/well) were differentiated to macrophages in 6-well plates for 24 h in 2 ml RPMI containing PMA. Media was replaced with RPMI without PMA and macrophages were incubated for an additional 24 h. Macrophages were then treated with indicated cytokines for 24 h. Cells in each well were lysed in ∼120 µl sample buffer (50 mM Tris HCl pH 6.8, 0.02% bromophenol blue, 12.5 mM EDTA, 2% SDS, 10% glycerol, 4% 2-mercaptoethanol) and boiled for 5 − 15 min.

A 20 µl volume of sample was loaded per well of polyacrylamide gel (4% stacking, 8% resolving) and proteins separated by SDS-PAGE (200 V, 50 min). Gels were blotted onto PVDF membranes overnight at 4 °C in Towbin buffer (30 V, ∼16 h). PVDF membranes were blocked for 1 h at room temperature in blocking buffer (PBS, 5% nonfat milk, 0.1% tween-20). Primary rabbit polyclonal anti-XIRP1 antibody (Thermo Fisher Scientific, PA5-53487) was added at a 1:5,000 dilution or mouse anti-β-actin [AC-15] (abcam, ab6276) at a 1:10,000 dilution and incubated at room temperature for 2 h with rocking. PVDF membranes were washed for 10 min three times in PBS-T (PBS, 0.1% tween-20). Secondary HRP-conjugated anti-mouse (GE Healthcare, NA931) or anti-rabbit (GE Healthcare, NA934) were added at 1:10,000 dilution for 1.5 h. PVDF membranes were washed for 10 min 3 times with PBS-T. To image blots, Clarity Max ECL Western Blotting Substrate (Bio-Rad laboratories, 1705062) was used with a ChemiDoc MP Imager System (Bio-Rad Laboratories).

### Immunofluorescence and cell staining

Cells were grown to desired conditions in 4-well chambered cover glass (Cellvis, C4-1.5H-N) or in Corning black 96-well clear bottom polystyrene microplates (Milliopore Sigma, CLS3904). Growth media was removed, and monolayers were washed with PBS (AmericanBio, Inc) prior to fixation with 4% PFA solution (Santa Cruz Biotechnology, Inc) for 20 min at room temperature. Fixed monolayers were washed three times, then incubated for 1 h in permeabilization buffer (PBS, 1% BSA, 0.1% saponin) at room temperature with rocking. The primary antibody was diluted in fresh permeabilization buffer based on manufacturers recommendation and cells were incubated overnight at 4 °C with rocking. Cells were washed (10 min) 3 times with PBS, the secondary antibody was added in fresh permeabilization buffer (1:2,000) for 2 h at room temperature in the dark with rocking. Cells were washed (10 min) 3 times with PBS and imaged within 24 h. To stain nuclei, either DAPI (Thermo Fisher Scientific, 62247) or Hoechst 33342 (Thermo Fisher Scientific, H3570) was added along with secondary antibodies. F-actin was stained with Phalloidin DyLight 650 (Thermo Fisher Scientific, 21838) during the incubation with secondary antibodies.

Dilutions of primary antibodies were as follows: mouse monoclonal anti-ARP3 [FMS338] (Millipore Sigma, A5979), 1:500; mouse monoclonal anti-LAMP1 [H4A3] (Abcam, ab25630), 1:100; rabbit polyclonal anti-MX1 (Thermo Fisher Scientific, PA5-22101), 1:250; monoclonal anti-Paxillin [Y113] (Abcam, ab32084), 1:250; rabbit polyclonal anti-VASP (Millipore Sigma, HPA005724), 1:400; mouse monoclonal anti-Vinculin [7F9] (Santa Cruz Biotechnology, Inc; sc-73614), 1:250; mouse monoclonal anti-Xinα [D-8] (Santa Cruz Biotechnology, Inc; sc-166658), 1:200; rabbit polyclonal anti-XIRP1 (Thermo Fisher Scientific, PA5-53487), 1:1,000; rabbit polyclonal anti-XIRP1 (Thermo Fisher Scientific, PA5-48605), 1:250. Secondary antibodies from Thermo Fisher Scientific were as follows: Alexa Fluor 488 donkey anti-mouse (A21202); Alexa Fluor 488 goat anti-rabbit (A11034); Alexa Fluor 568 goat anti-mouse (A11004); Alexa Fluor 568 donkey anti-rabbit (A10042).

### Microscopy, image processing, and fluorescence measurements

Samples in clear-bottom 96-well plates were imaged using an ImageXpress Pico Automated Cell Imaging System (Molecular Devices) or a Cytation C10 Confocal Imaging Reader (Agilent Technologies). Samples in 4-well chambered cover glass were imaged with either a Nikon epifluorescence microscope, Nikon TiE inverted spinning disk confocal, or a DeltaVision OMX SR Imaging System (GE Healthcare). Reconstruction of super-resolution images was done with softWoRx (GE Healthcare). Live-cell microscopy images were acquired using an environmental chamber (37 °C, 5% CO_2_) with the widefield mode of the DeltaVision OMX SR Imaging System. Live-cell images were deconvoluted with softWoRx. Adjustments to image brightness and contrast was applied to entire images using Fiji/ImageJ 1.53c (Wayne Rasband, National Institutes of Health, USA).

For quantification of MX-1 and XIRP1 immunostaining in fibroblast, total fluorescence was measured from full 20x microscopy fields using the Gen5 Image Prime Software (Agilent Technologies); due to the higher non-specific signal of the mouse anti-XIRP1 antibody on macrophage nuclei, the proportion of macrophages with XIRP1+ podosomes was determined using Fiji software. In other podosome measurements, the Fiji-based macro Poji^24^ was used to measure fluorescence intensity of individual podosomes and to determine podosome number per cell. Confocal microscopy was used to acquire Z-stacks at the base of podosomes marked by vinculin. For consistency, the Z-slice with the highest vinculin signal was selected for measurements associated with phalloidin staining of F-actin.

### Statistical analysis

Statistical methods were selected based on the distribution and type of data (parametric or non-parametric; paired or unpaired). Unless otherwise indicated, GraphPad Prism was used to perform statistical analysis on a sample size of n ≥ 3. The specific statistical method tested and summary statistics are indicated in the figure captions.

## Results and discussion

### IFN-γ signaling induces hundreds of actin-related genes in humans

To identify actin-associated genes responsive to IFN-γ signaling, we combined whole cell RNA-sequencing (RNA-Seq) with computational retrieval in both immune and non-immune human cell populations. Fibroblasts served as a non-immune cell source given their broad tissue distribution and robust IFN-γ-dependent response.^25^^,^^26^ Here primary human derived Hs27 fibroblasts were chosen for RNA-Seq analysis because they form flattened polygonal sheets with multiple intercellular contacts for pathogen dissemination and their non-cancerous phenotype. In parallel, ARCHS^4^ data-mining^20^ was used to interrogate RNA-Seq profiles of IFN-γ-activated human monocyte-derived macrophages (HMDMs) and THP-1 differentiated macrophages as immune phagocytic models. THP-1 cells are also amenable to CRISPR-Cas9 engineering for later functional studies.

**Figure 1:**
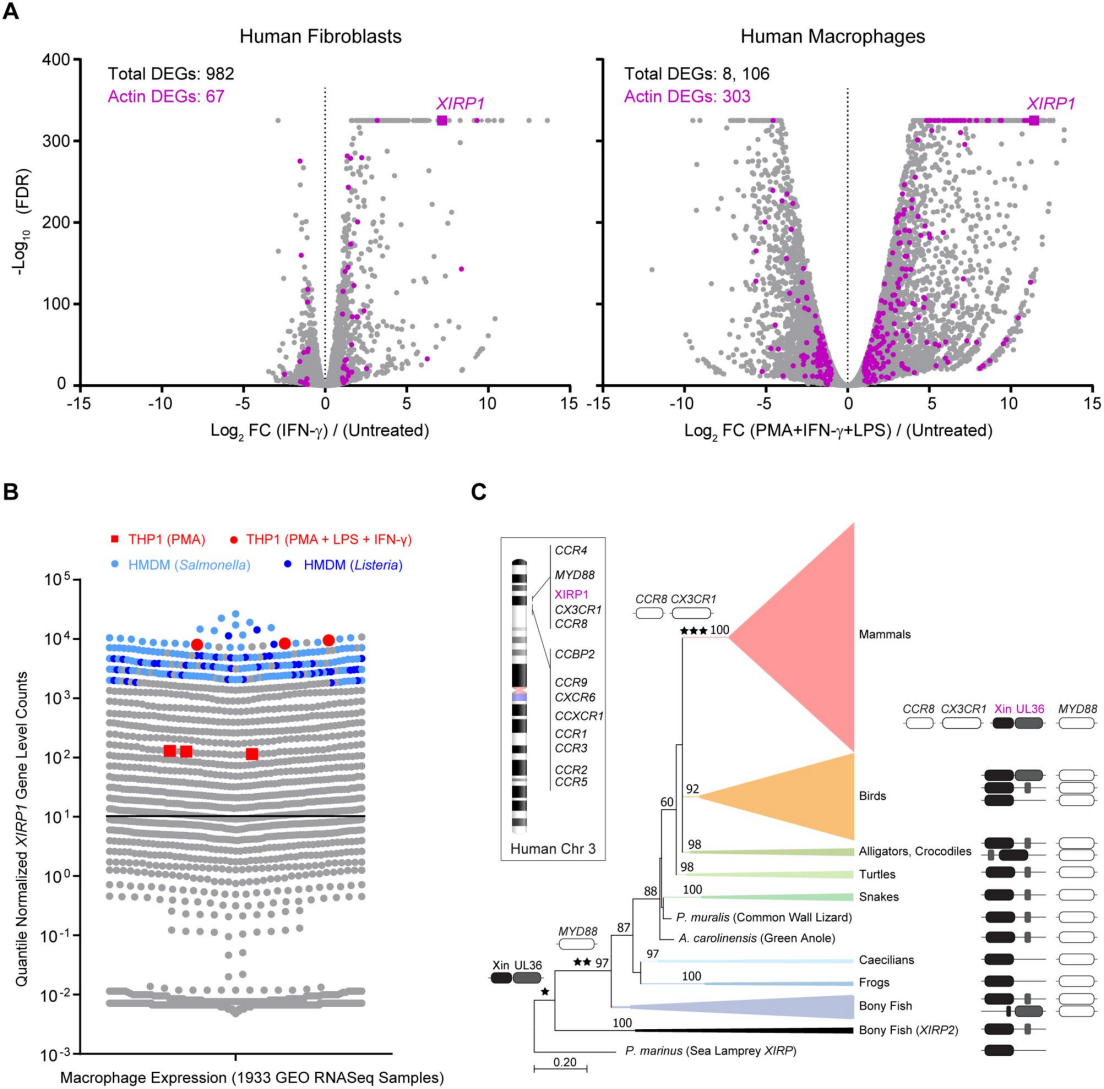
RNA-Seq and *in silico* analysis identify *XIRP1* and actin-associated genes regulated by IFN-γ. (A) Volcano plots depicting RNA-Seq analysis of Hs27 fibroblasts treated for 24 h with IFN-γ (1,000 U/ml) compared to untreated cells and re-analysis of classically activated THP-1 macrophages (PMA differentiated, LPS and IFN-γ treated) compared to untreated THP-1 monocytes (re-analyzed GEO datasets: GSM3729256-9, GSM3729250-2; PMID: 32449925). *XIRP1* and other actin-related DEGs are shown in magenta among overall DEGs. *edgeR exact test*; FDR, 5% false discovery rate-adjusted *P* value; FC, fold change. (B) *XIRP1* gene-level counts across human macrophage RNA-Seq data sets obtained from ARCHS^4^. Samples from THP-1 derived macrophages are shown in red and from human monocyte derived macrophages are shown in blue (*Salmonella-*infected, light blue; *Listeria*-infected, dark blue). Bar shown indicates median (C) Maximum-likelihood tree with JTT matrix-based model shows the distribution of *XIRP1* across vertebrates. Shown are full or partial Xin and UL36 domains identified through the conserved domain database. The presence of *MYD88* and genes encoding macrophage chemokine receptors within 1,000,000 bp region flanking *XIRP1* is indicated. Additional genes within two chemokine receptor clusters in human chromosome 3 are shown.

RNA-Seq of Hs27 fibroblasts detected 982 differentially expressed genes (DEGs) (fold-change ≥ 2, 5% false discovery rate-adjusted *P* value < 0.05) after IFN-γ treatment. Of these DEGs, 645 (∼65.7%) were upregulated and 337 (∼34.3%) downregulated (Fig. 1A). Among the IFN-γ-induced genes were known cell-autonomous immunity proteins (*GBP1-6*, *IDO1*, *APOL1-4, 6);*^1^^,^^8^^,^^26^ RNA sensors of the oligoadenylate synthase family (*OAS1-3, OASL*); chemokines (*CXCL9-11, CX3CL1*), and proteins involved in antigen presentation (*HLA-DOA, HLA-DRA, HLA-DRB1)*. Gene Ontology (GO) classification identified 67 DEGs (∼6.8% of the total) with actin-related functions (Fig. 1A). They included the anti-viral protein tetherin (*BST2*), the monocyte/macrophage chemoattractant CX3CL1, and a poorly characterized actin-binding protein, XIRP1 (Tables S1 and S2).

We chose XIRP1 for further analysis because it lacks any known role in host defense, has actin-binding activity, and was markedly induced (∼143.3-fold) in our RNA-Seq data. *XIRP1* was also among the most elevated ISGs in 303 DEGs uncovered in THP-1 cells and pronounced *XIRP1* expression was observed by ARCHS^4^ comparison of gene-level counts from 1,933 macrophage RNA-Seq datasets in the GEO database (Fig. 1B). Within these data sets, HMDMs infected with *Listeria* or *Salmonella* and M1 macrophage polarization using IFN-γ plus lipopolysaccharide (LPS) had some of the highest normalized counts (Fig. 1B). Indeed, XIRP1 was the highest induced actin-binding protein (∼2,745.8-fold) when THP-1 monocytes are compared to M1 polarized THP-1 macrophages in our computational pipeline (GEO data sets: GSM3729250-2 vs GSM3729256-58; PMID 32449925) (Fig. 1A).

### Human *XIRP1*: a novel ISG enhanced by IL-1β or bacterial signals

The profound induction by IFN-γ was unexpected since XIRP1 homologs are only known to have cardiac and musculoskeletal functions.^27–29^ Phylogenetic and gene synteny analysis of regions flanking the XIRP1 locus, however, identified linkages with *MYD88* across numerous vertebrates (Fig. 1C); this gene encodes an innate immune adapter critical for toll-like receptor (TLR) and IL-1β signaling.^30^ Within mammals, *XIRP1* was also surrounded by chemokine receptor genes important for macrophage chemotaxis (ie *CX3CR1*, *CCR8*). Indeed, both the *XIRP1* and *MYD88* loci reside within 2 chemokine receptor clusters on human chromosome 3. When considering XIRP1 domain composition, differences in the number of xin repetitive regions exist between mammals and other species. While ∼17 of these F-actin binding domains are found in humans and other mammalian orthologs, over 26 repetitive domains exist in most species ranging from birds to fish.^28^ This dramatic reduction in mammalian xin domains suggests XIRP1 may have roles beyond its ancestral muscle-related activities.

**Figure 2:**
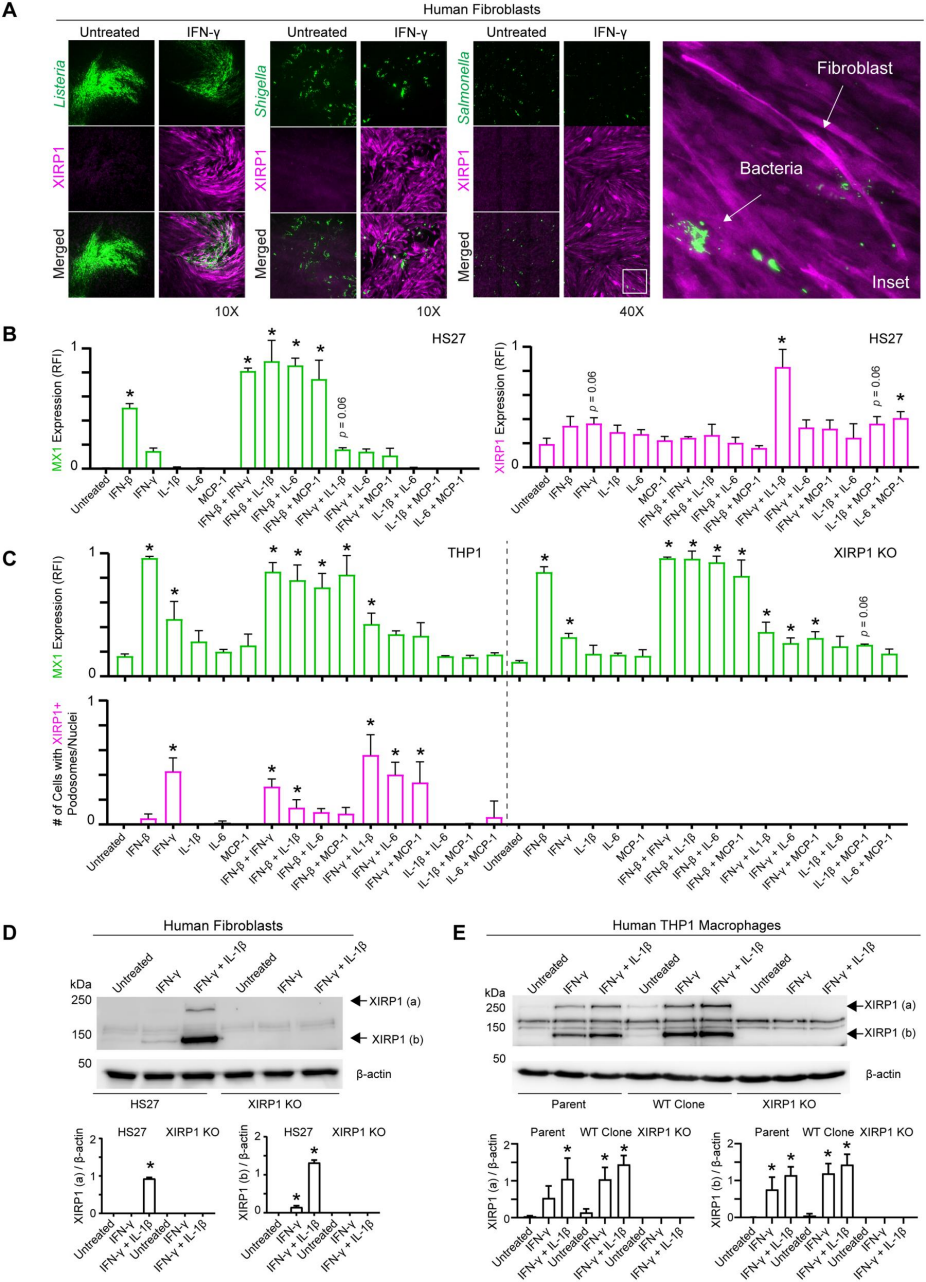
Human XIRP1 expression is dependent on IFN-γ and other immune stimuli. (A) Immunostaining of Hs27 fibroblast monolayers infected with GFP-expressing *Listeria* (left) or *Shigella* (middle) in the presence or absence of 1,000 U/ml IFN-γ. Normal human intestinal myofibroblasts monolayers infected with GFP-expressing *Salmonella* (right) at the indicated IFN-γ concentrations. (B) Immunostaining quantification of Hs27 fibroblast monolayers and (C) THP-1 derived macrophages 24 h post-treatment with the indicated cytokines; fluorescent signal from full fibroblast fields was quantified or fraction of macrophages showing signal beyond the nucleus. (D) XIRP1 Immunoblots from Hs27 fibroblasts and (E) THP-1 derived macrophages 24 h post-treatment with indicated cytokine. One-way ANOVA with Dunnett’s multiple comparisons; *P < 0.05; means with standard deviation are shown.

This idea was reinforced by immunostaining and immunoblotting for endogenous XIRP1 in response to immune and infectious stimuli; here, the generation of CRISPR-Cas9 stable *XIRP1* deletions (XIRP1 KO) in Hs27 fibroblasts and THP-1 macrophages confirmed antibody specificity. IFN-γ-treated fibroblast monolayers infected with diverse pathogens (*Listeria*, *Shigella*, and *Salmonella*) had robust XIRP1 expression (Fig. 2A), whereas treatment with IFN-γ, IL-1β, or IL-6 alone did not (Fig. 2B and D), despite high mRNA levels in our RNA-Seq analysis. Additional infection-triggered signals therefore appear necessary for full IFN-γ-dependent XIRP1 protein expression. Screening 15 human cytokine treatments found IFN-γ plus IL-1β yielded the highest XIRP1 protein levels, suggesting that STAT1 and NF-κB signals converge on the *XIRP1* promoter (Fig. 2 and Fig. S1). In human THP-1 macrophages, IFN-γ appeared sufficient for robust XIRP1 expression (Fig. 2C and E); however, phorbol myristate acetate (PMA)-treatment during differentiation probably induces NF-κB autocrine IL-1β release (Table S2).^31^^,^^32^ Notably, type I IFN-β did not induce XIRP1expression despite stimulating MX1 as a positive control (Fig. 2B, C and Fig. S1). Thus, *XIRP1* is primarily a type II IFN-induced gene that benefits from NF-κB signals for optimal protein expression in both immune and non-immune human cells.

### IFN-γ-induced XIRP1 localizes to the actin-rich podosomal network

**Figure 3:**
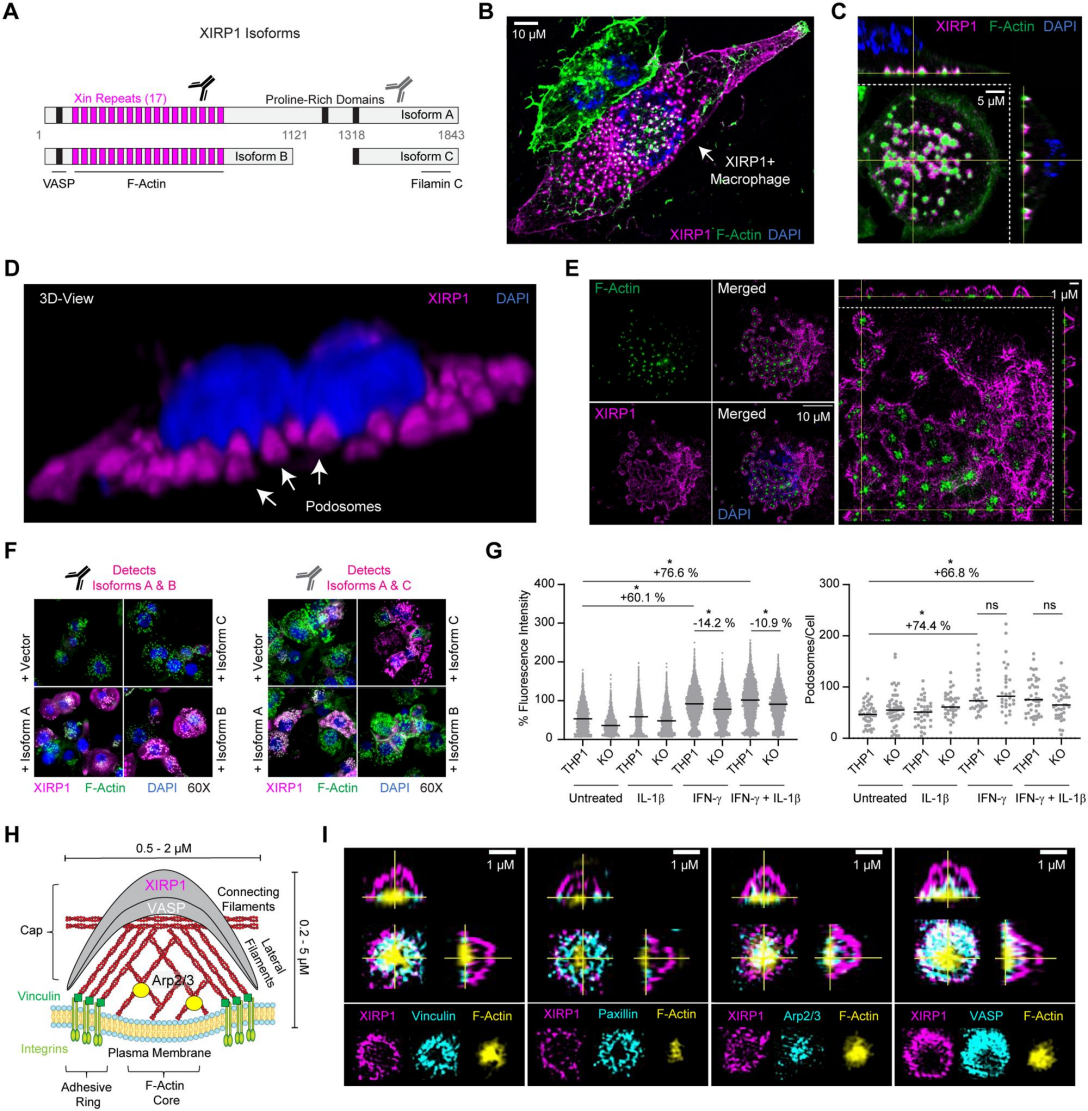
XIRP1 is a component of podosomes in IFN-γ-activated macrophages. (A) Diagram of the three human XIRP1 isoforms shows the actin-binding xin repeat domains, proline-rich domains and selected XIRP1 regions previously shown to interact with VASP, F-actin, and Filamin C. The epitopes detected by the anti-XIRP1 antibodies are indicated. (B) A representative single z-slice confocal microscopy image of immunostained THP-1 macrophages shows XIRP1 localization to sub-cellular podosomes (magenta and green specks). (C) orthogonal views and (D) 3D rendering of confocal microscopy images show dozens of conical XIRP1 structures distributed at the base of macrophages. (E) Super-resolution structured-illumination microscopy (SIM) of a single cell immunostained for XIRP1 (left) and orthogonal views of the macrophage podosome network (right). (F) Epi-fluorescent microscopy images of XIRP1 KO macrophages expressing individual isoforms or vector alone upon retroviral transduction. Immunostaining with isoform-specific antibodies shows podosome localization of individual XIRP1 isoforms. (G) Measurement of F-actin fluorescence intensity in podosomes (n ≥ 1,884) and number of podosomes per cell (n ≥ 30 cells) in THP-1 macrophages and XIRP1-knockout cells. Two-way ANOVA with Tukey’s multiple comparisons; *P < 0.05; medians are shown. (H, I) Super-resolution SIM of single podosomes immunostained for XIRP1 and indicated podosome markers. Orthogonal views are shown (top) along with individual channels from a single z-slice through the base of the podosome (bottom). The model shows the localization of XIRP1 extending from the podosome cap to the adhesion ring along the lateral actin filaments. Dimensions on the model are based on approximations previously reported.

Next, we examined where endogenous XIRP1 resides in human cells. Immunostaining IFN-γ-activated human macrophages found XIRP1 was organized in a bespeckled pattern at the base of cells near the underlying substrate (Fig. 3A–C). These XIRP1 clusters resembled actin-rich adhesion foci termed podosomes.^33^ Indeed, XIRP1 forms a unique toroidal pattern around a central F-actin core (Fig. 3B–C and E). The prototypic conical shape of podosomes was revealed in orthogonal views and 3D image reconstructions (Fig. 3C and D).

Human XIRP1 exists as 3 alternatively spliced protein isoforms varying in length and domain composition (Fig. 3A).^27^ We therefore re-introduced isoforms A (a.a. 1–1,843), B (a.a. 1–1,121), or C (a.a. 1,318–1,843) into XIRP1 KO human macrophages via retroviral transduction to determine if each can assemble into podosomes. While transduction with the shorter isoforms B and C was more efficient than full-length isoform A (Fig. 3F, lower-left panels), all 3 were recruited to podosomes as shown using isoform-specific antibodies (Fig. 3F). Isoforms A and B contain xin repeat domains, which directly bind F-actin, as well as a proline-rich domain that physically interacts with the actin-binding protein VASP;^34^ isoform C does not contain xin repeats but does include domains that engage Filamin C and other actin-binding proteins.^34^ It is therefore likely that different XIRP1 isoforms are recruited through both actin-binding xin repeats and other domains that engage podosome accessory proteins.

Super-resolution structured illumination microscopy (SIM) verified XIRP1 encapsulation of the F-actin core at the base of individual podosomes and throughout the podosome network (Fig. 3E). Within this nanoarchitecture, XIRP1 forms a dome-shaped cap that extends down into the adhesion ring composed of vinculin and paxillin (Fig. 3H and I). Because XIRP1 is excluded from the branched F-actin core containing Arp2/3 and instead is closely associated with the bundling protein, VASP, the precise location of XIRP1 seems to extend along lateral, linear actin filaments. These lateral filaments link podosomes together as a mechanosensitive unit and add stiffness to the F-actin core.^33^ Indeed, XIRP1 KO macrophages had significant reductions in podosome F-actin content but not podosome number after IFN-γ treatment (Fig. 3G). Thus, encapsulation of actin-rich bundles by XIRP1 appears to stabilize pre-existing podosomes and the tethers between them rather than instigating *de novo* construction.

### IFN-γ-induced XIRP1 encapsulates actin-utilizing bacteria within macrophages

The striking localization of XIRP1 to the actin-rich podosomal network and its encapsulating behavior led us to examine human macrophages infected with actin-utilizing pathogens. We chose *Listeria monocytogenes* as a well-studied Gram-positive bacteria that escapes its endolysosomal compartment to engage cytosolic actin for intracellular motility and subsequent dissemination.^35^ The ActA virulence factor of *L. monocytogenes* recruits the ARP2/3 complex plus VASP to polymerize host actin and propel bacteria.^36^ Since XIRP1 and VASP lie adjacent to each other in macrophage podosomes (Fig. 3H and I) and interact based on domain architecture,^29^ we hypothesize that XIRP1 is recruited along with VASP onto *Listeria*. Here it could stiffen actin filaments to impact bacterial spread as part of the IFN-γ-induced defense program.

**Figure 4:**
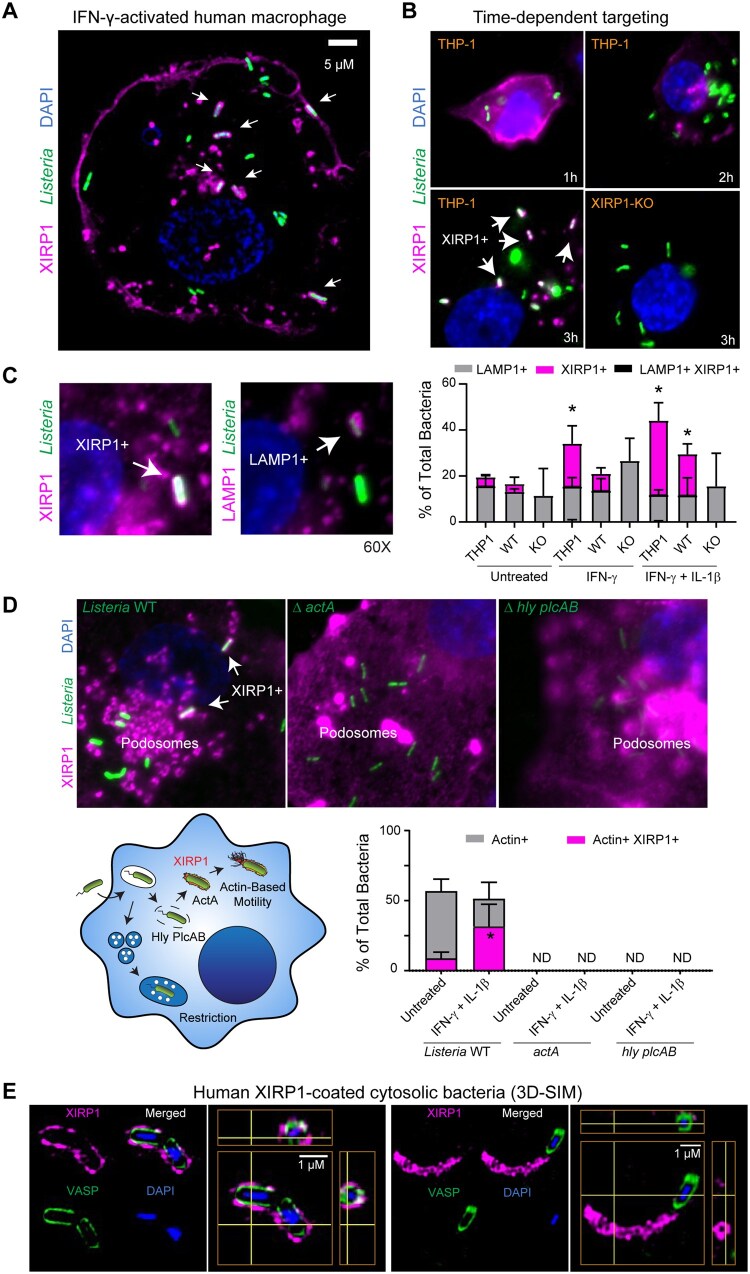
XIRP1 is recruited to the surface of intracellular *Listeria*. (A) Confocal microscopy of THP-1 macrophages infected with GFP-expressing *Listeria* (single z-slice, 3 h post-infection). (B) Epifluorescence microscopy of infected THP-1 and XIRP1-knockout macrophages at indicated time points post-infection. (C) Representative image and quantification of lysosomal LAMP1 and XIRP1-coated bacteria. Cytokine-activated macrophages showed high levels of cytosolic bacteria (LAMP1-negative) targeted by XIRP1. (D) Immunostaining of THP-1 macrophages infected with wildtype *Listeria* or mutants deficient in actin-based motility (*actA*) or phagosome rupture (*hly plcAB*). Model shows *Listeria* invasion of the macrophage cytoplasm and actin-based motility when escaping XIRP1. (E) Super-resolution structured-illumination microscopy of replicating (left panels) and motile (right panels) *Listeria* in THP-1 macrophages (single channels, left; orthogonal views, right). One-way ANOVA with Dunnett multiple comparisons; *P < 0.05; means with standard deviation are shown; ND, not detected.

We found XIRP1 directly targeted and encapsulated GFP-expressing *L. monocytogenes* in IFN-γ-activated human macrophages (Fig. 4A and B). This occurred at later rather than early time points post-infection when most *Listeria* have already ruptured their vacuole and escaped into the cytosol. Indeed, XIRP1-positive bacteria lacked the lysosomal marker LAMP1, while LAMP1-positive bacteria did not co-localize with XIRP1 (Fig. 4C). Moreover, *Listeria* mutants devoid of listeriolysin plus phospholipase A and B (*hlyplcAB*) needed for vacuolar rupture^35^ confirmed the phenotype; this triple mutant failed to recruit XIRP1 (Fig. 4D). Similarly, an *actA* mutant which accesses the cytosol but cannot recruit host actin^35^ also failed to colocalize with XIRP1 (Fig. 4D). Together these results show human XIRP1 assembles on cytosolic *Listeria* in an ActA-dependent manner.

Super-resolution SIM and live widefield microscopy found replicating and individual bacteria were both targeted by XIRP1 (Fig. 4E and S1–3 Videos). It formed an outer coat surrounding VASP on the bacterial surface (Fig. 4E, left). Notably, fully encased bacilli appeared less motile and, in some cases, became immobilized (S1–3 Video). However, when XIRP1 coating was incomplete or separated from the underlying VASP complex, *L. monocytogenes* escaped, leaving behind XIRP1 as a hollow tube while VASP remained attached to the bacterial surface (Fig. 4E, right; S3 Video). Hence the behavior of VASP differs from XIRP1 during bacterial spread. A stiffer actin-based coat surrounding *Listeria* when XIRP1 is present may limit motility or serve as an innate immune signaling platform like the recently described human GBP defense complex assembled on cytosolic *Salmonella.*^15^^,^^16^ Future work will delineate both possibilities plus the downstream consequences of bacterial encapsulation in IFN-γ-activated human cells. In sum, these studies have identified a new actin-binding ISG–XIRP1 that stabilizes podosomes and assembles on bacterial pathogens during the cell-autonomous immune response to infection.

## Supplementary Material

vkag116_Supplementary_Data

## Figures and Tables

**Table:** 

Funder	Grant reference number	Author
Howard Hughes Medical Institute Howard Hughes Medical Institute	GT11834 GT16788	Rodolfo Urbano Rodolfo Urbano
National Institutes of Health	5R01 AI108834-12	John D. MacMicking
National Institutes of Health	3R01 AI068041-15	John D. MacMicking

## Data Availability

All published data shall be available freely. Materials will be available under an MTA. No new software is developed or used.
