# Supplementary material for: Human XIRP1 is a new podosome protein targeting cytosolic bacteria as part of the IFN-γ defense program
Source: J Immunol. 2026 Jun 15;215(6):vkag116. doi: 10.1093/jimmun/vkag116 (PMC13267782; doi:10.1093/jimmun/vkag116)
Supplement: vkag116_Supplementary_Data [file vkag116_supplementary_data.zip › Supp_Video_Captions.pdf]

Supplementary Videos

S1 Video
XIRP1 coats intracellular *Listeria* in THP-1 macrophages
Live-cell widefield microscopy of THP-1 macrophages expressing XIRP1B-mCherry ~2.5 h post-infection with GFP-expressing *Listeria.* Bar: 10 mm

S2 Video
***Listeria* escapes the XIRP1 coat in THP-1 macrophages**
Live-cell widefield microscopy of THP-1 macrophages expressing XIRP1B-mCherry ~2.5 h post-infection with GFP-expressing *Listeria.* Bar: 10 mm

S3 Video
***Listeria* remain or escape the XIRP1 coat in THP-1 macrophages**
Live-cell widefield microscopy of THP-1 macrophages expressing XIRP1B-mCherry ~2.5 h post-infection with GFP-expressing *Listeria.* Arrows indicate bacteria that escape or remain in the XIRP1 coat. Bar: 10 mm.
