# Supplementary material for: Human XIRP1 is a new podosome protein targeting cytosolic bacteria as part of the IFN-γ defense program
Source: J Immunol. 2026 Jun 15;215(6):vkag116. doi: 10.1093/jimmun/vkag116 (PMC13267782; doi:10.1093/jimmun/vkag116)
Supplement: vkag116_Supplementary_Data [file vkag116_supplementary_data.zip › S1_Fig.pdf]

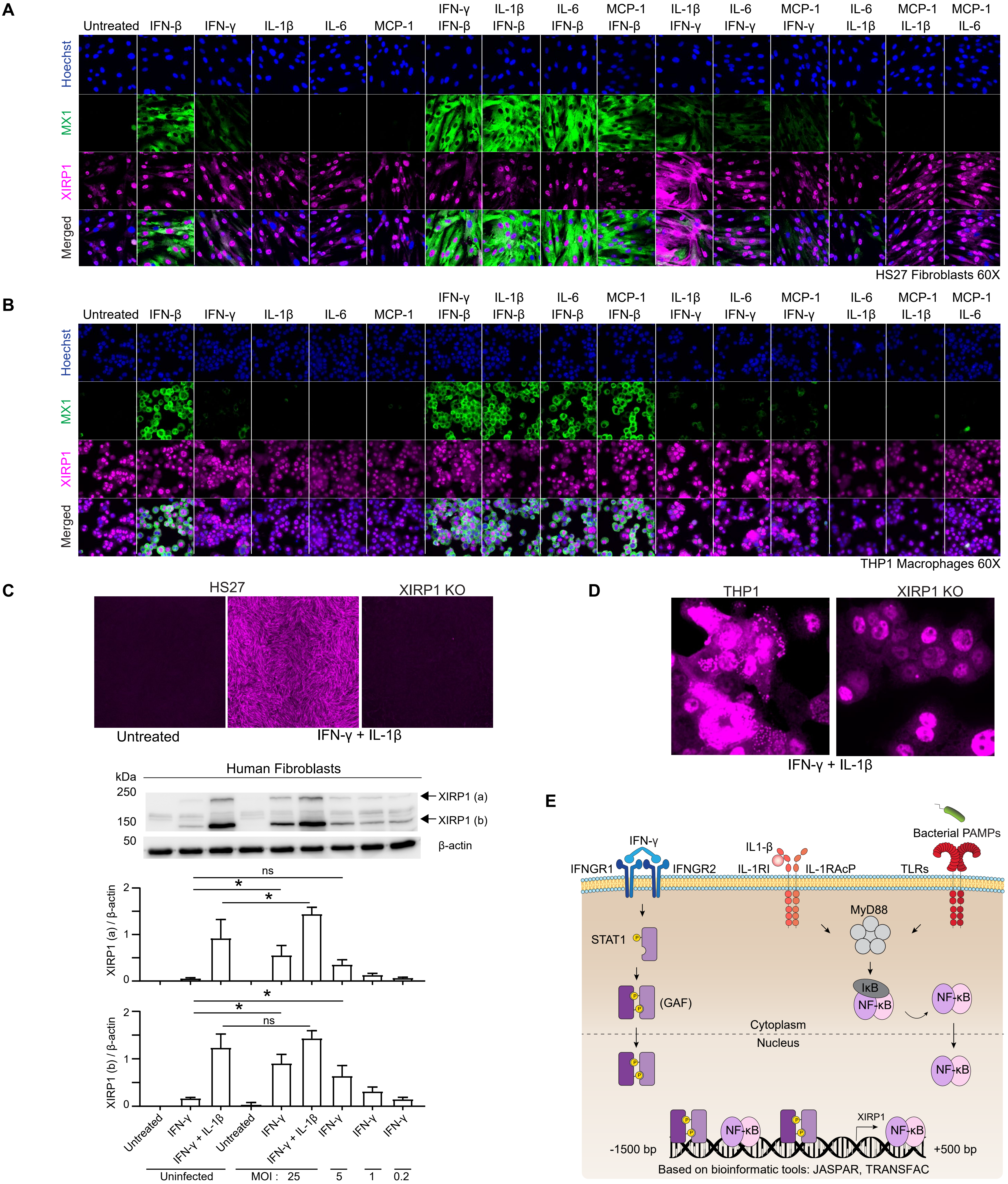

S1 Fig. Stimulation of XIRP1 by different cytokines and *Listeria*

Representative immunostaining images of (A) fibroblasts and (B) macrophages used in the quantification of XIRP1 and MX1 expression. (C) Immunostaining (top) showing specificity of anti-XIRP1 antibody used in fibroblast infections. Immunoblots (bottom) showing enhanced XIRP1 expression in *Listeria*-infected HS27 cells treated with IFN-γ. One-way Anova with Tukey's multiple comparisons test; *$p < 0.05$; means with standard deviation are shown. (D) Representative image of XIRP1 staining in macrophages showing cytoplasmic staining of XIRP1 and non-specific nuclear signal. (E) Model shows immune pathways that stimulate XIRP1 expression and putative transcription factor binding-sites on the XIRP1 regulatory region (bioinformatically predicted with indicated webtools).
