# Supplementary material for: Human XIRP1 is a new podosome protein targeting cytosolic bacteria as part of the IFN-γ defense program
Source: J Immunol. 2026 Jun 15;215(6):vkag116. doi: 10.1093/jimmun/vkag116 (PMC13267782; doi:10.1093/jimmun/vkag116)
Supplement: vkag116_Supplementary_Data [file vkag116_supplementary_data.zip › S2_Fig.pdf]

A

interferome.org TRANSFAC TF Analysis

Gene Name	Site	Core Match	Matrix Match	Start Site	End Site
**XIRP1**					
	NFKAPPAB50	1	0.885	1702	1712
	NFKAPPAB50	0.856	0.918	184	194
	STAT	1	0.992	626	639
	STAT1	1	0.996	61	71
	STAT1	1	0.965	52	74
	STAT1	0.968	0.971	615	637
	STAT1	0.76	0.843	56	77
	STAT1	0.76	0.848	619	640
	STAT3	1	0.956	620	636
	STAT3	1	0.93	57	73
**Xirp1**					
	IRF1	1	1	1940	1947
	IRF8	1	1	1937	1944
	STAT1	1	0.998	635	643

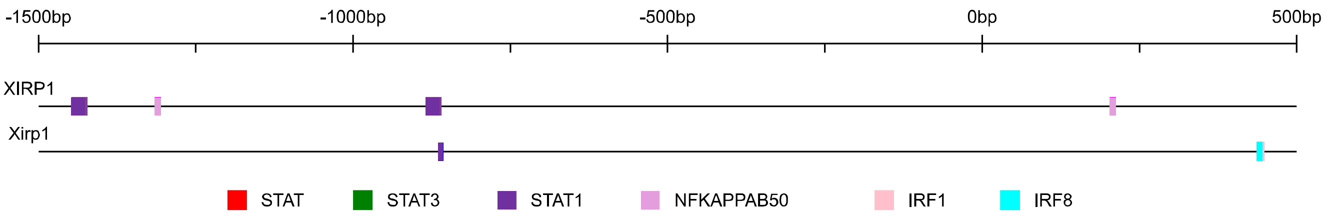

B

genome.ucsc.edu JASPAR TF Analysis

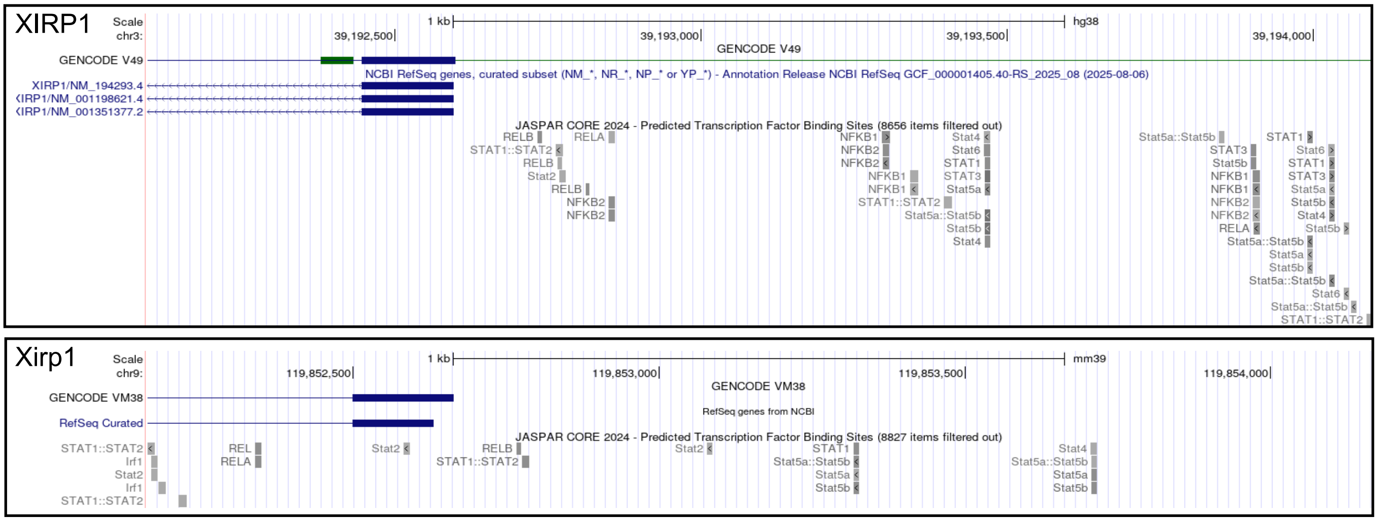

shade									
score in range	≤ 166	167-277	278-388	389-499	500-611	612-722	723-833	834-944	≥ 945

Conversion table:

**Item score**	0	100	131	200	300	400	500	600	700	800	900	1000
**p-value**	1	0.1	0.049	10^{-2}	10^{-3}	10^{-4}	10^{-5}	10^{-6}	10^{-7}	10^{-8}	10^{-9}	$\leq 10^{-10}$

S2 Fig. Predicted Binding Sites of Selected Transcription Factors

(A) TRANSFAC-based prediction of transcription factor (TF) binding sites using interferome.org default settings for human (XIRP1) and mouse (Xirp1) promoter regions. (B) JASPAR-based predicted TF binding sites using the UCSC genome browser. The displayed selection sites were based on scores >350 for IRF, STAT, and NFκB TF families.
